# Supplementary figures and images for: Multiple Stressors in the Anthropocene: Urban Evolutionary History Modifies Sensitivity to the Toxic Effects of Crude Oil Exposure in Killifish
Source: Evol Appl. 2025 May 15;18(5):e70112. doi: 10.1111/eva.70112 (PMC12081835; doi:10.1111/eva.70112)

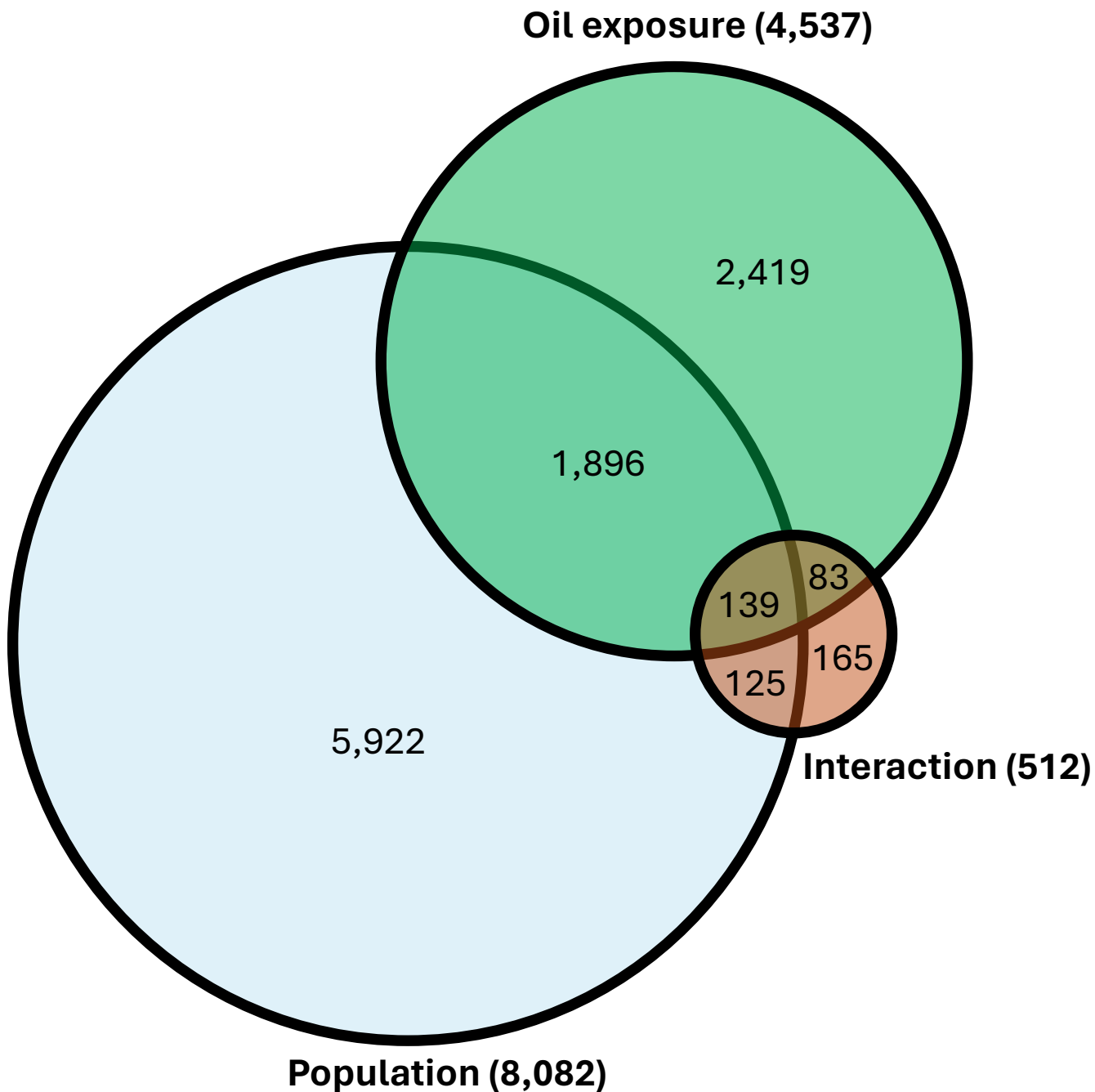

Supplement: Supplementary file 1 — Figure S1. Numbers of differentially expressed genes (FDR adj. p < 0.05) for the main effects of population and oil exposure concentration treatment groups, and their interaction. Figure S2. Gene Ontology (GO) enrichment analyses for the 512 genes that showed an oil exposure response that varied between populations (significant oil dose‐by‐population interaction, p < 0.05). Cluster 1 represents functions enriched in the top cluster of DEGs in 5a (genes upregulated in response to oil exposure in sensitive populations), and Cluster 2 represents functions enriched in the bottom cluster of DEGs in 5a (genes downregulated in response to oil exposure in sensitive populations). Figure S3. 4109 DEGs show a conserved transcriptional response to oil exposure in all four populations. We included genes with significant oil exposure response (FDR adj. p < 0.05) and excluded DEGs with a significant interaction effect of population by oil concentration (FDR adj. p < 0.1). (A) Heatmap showing parallel (conserved) transcriptomic responses to oil between populations. The four panels, left to right, show oil concentration‐responsive genes for the LA‐Reference, LA‐Polluted, TX‐Reference, and Tx‐Polluted populations, respectively. Within each population panel, increasing oil concentrations are organized starting from no‐oil controls (Con) on the left to the highest concentration of oil on the right. Individual genes are the rows. Genes (rows) were hierarchically clustered (Pearson correlation). Higher and lower transcript abundance is indicated in yellow and blue, respectively. Color intensity relates to fold‐increase or decrease of log2 expression (see color scale bar). (B) The first two principal components for the oil exposure‐responsive genes that were conserved in their response between populations. Arrows indicate the trajectory of gene expression change with increasing dose for each population. The base of each arrow represents the no‐oil control condition for that population, wh [file EVA-18-e70112-s002.zip › FigS1.pdf]

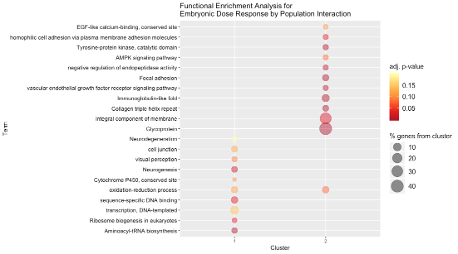

Supplement: Supplementary file 1 — Figure S1. Numbers of differentially expressed genes (FDR adj. p < 0.05) for the main effects of population and oil exposure concentration treatment groups, and their interaction. Figure S2. Gene Ontology (GO) enrichment analyses for the 512 genes that showed an oil exposure response that varied between populations (significant oil dose‐by‐population interaction, p < 0.05). Cluster 1 represents functions enriched in the top cluster of DEGs in 5a (genes upregulated in response to oil exposure in sensitive populations), and Cluster 2 represents functions enriched in the bottom cluster of DEGs in 5a (genes downregulated in response to oil exposure in sensitive populations). Figure S3. 4109 DEGs show a conserved transcriptional response to oil exposure in all four populations. We included genes with significant oil exposure response (FDR adj. p < 0.05) and excluded DEGs with a significant interaction effect of population by oil concentration (FDR adj. p < 0.1). (A) Heatmap showing parallel (conserved) transcriptomic responses to oil between populations. The four panels, left to right, show oil concentration‐responsive genes for the LA‐Reference, LA‐Polluted, TX‐Reference, and Tx‐Polluted populations, respectively. Within each population panel, increasing oil concentrations are organized starting from no‐oil controls (Con) on the left to the highest concentration of oil on the right. Individual genes are the rows. Genes (rows) were hierarchically clustered (Pearson correlation). Higher and lower transcript abundance is indicated in yellow and blue, respectively. Color intensity relates to fold‐increase or decrease of log2 expression (see color scale bar). (B) The first two principal components for the oil exposure‐responsive genes that were conserved in their response between populations. Arrows indicate the trajectory of gene expression change with increasing dose for each population. The base of each arrow represents the no‐oil control condition for that population, wh [file EVA-18-e70112-s002.zip › FigS2.png]

A.

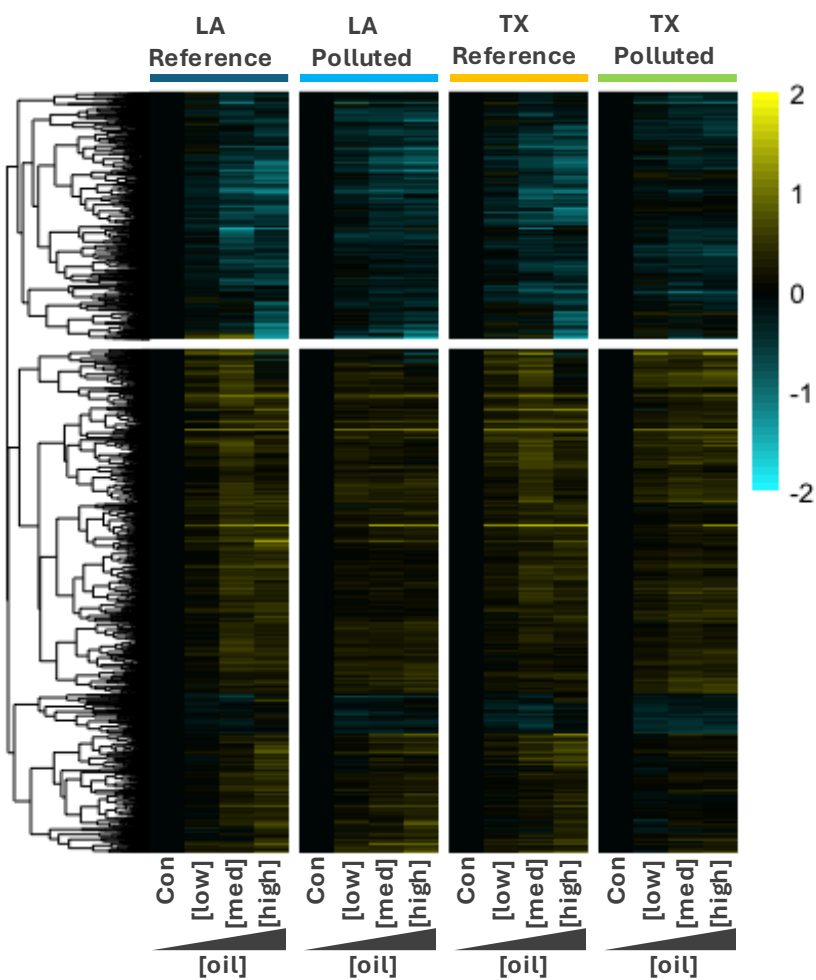

B.

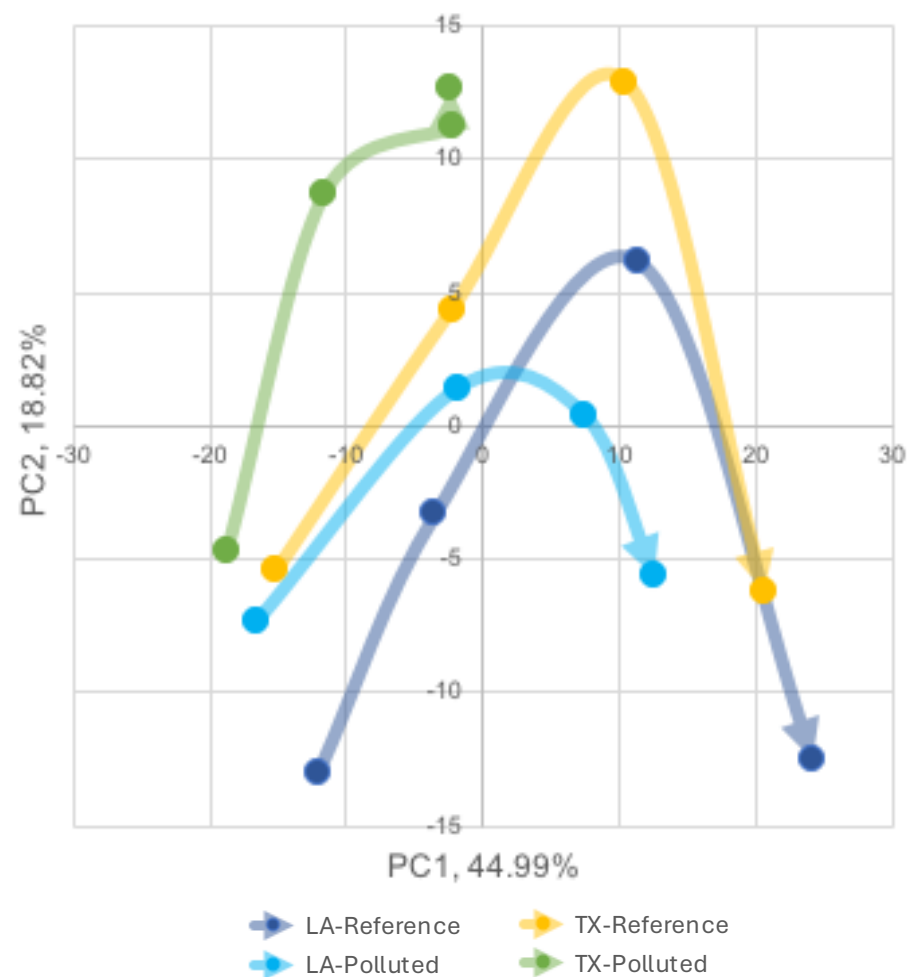

C.

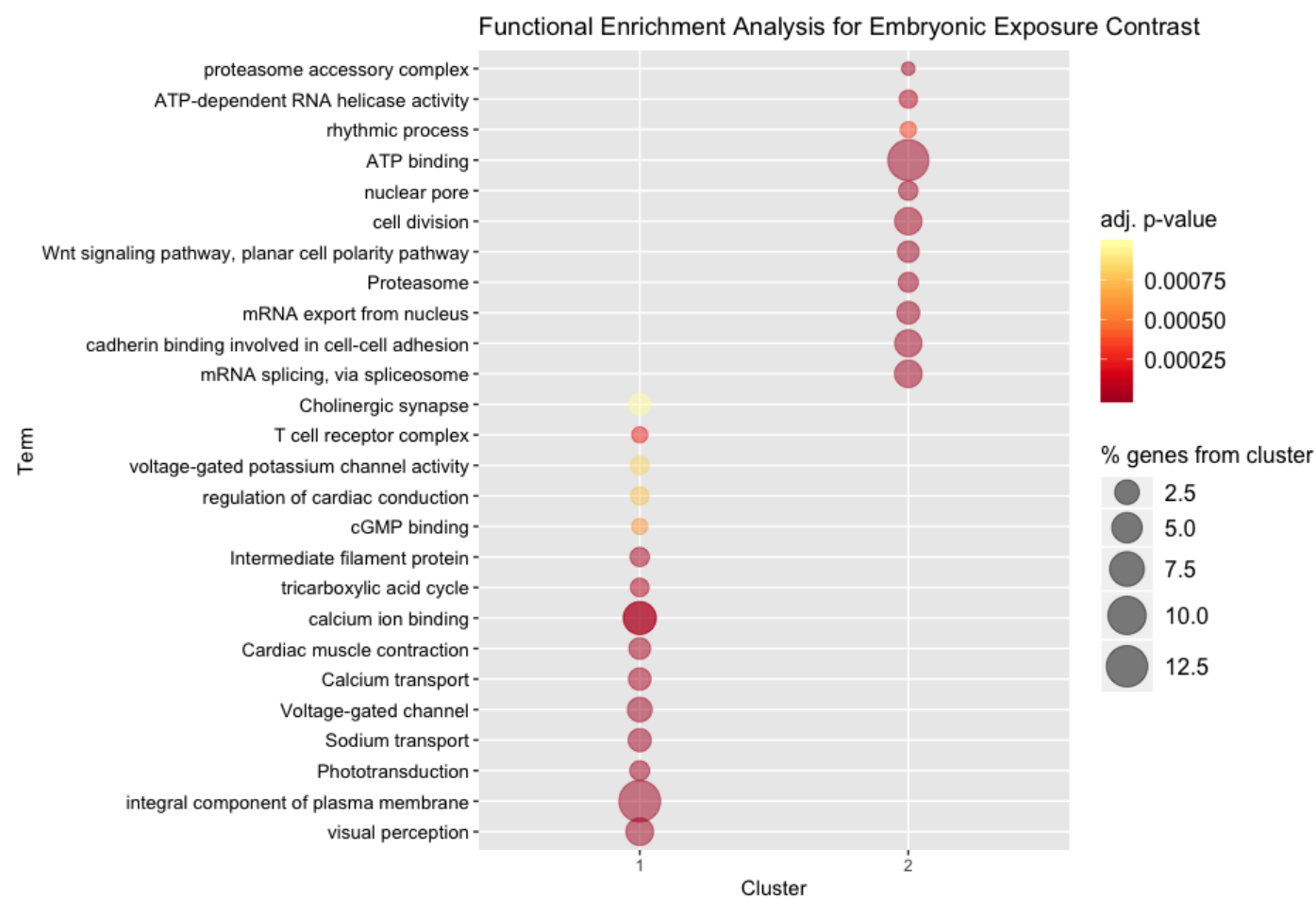

Supplement: Supplementary file 1 — Figure S1. Numbers of differentially expressed genes (FDR adj. p < 0.05) for the main effects of population and oil exposure concentration treatment groups, and their interaction. Figure S2. Gene Ontology (GO) enrichment analyses for the 512 genes that showed an oil exposure response that varied between populations (significant oil dose‐by‐population interaction, p < 0.05). Cluster 1 represents functions enriched in the top cluster of DEGs in 5a (genes upregulated in response to oil exposure in sensitive populations), and Cluster 2 represents functions enriched in the bottom cluster of DEGs in 5a (genes downregulated in response to oil exposure in sensitive populations). Figure S3. 4109 DEGs show a conserved transcriptional response to oil exposure in all four populations. We included genes with significant oil exposure response (FDR adj. p < 0.05) and excluded DEGs with a significant interaction effect of population by oil concentration (FDR adj. p < 0.1). (A) Heatmap showing parallel (conserved) transcriptomic responses to oil between populations. The four panels, left to right, show oil concentration‐responsive genes for the LA‐Reference, LA‐Polluted, TX‐Reference, and Tx‐Polluted populations, respectively. Within each population panel, increasing oil concentrations are organized starting from no‐oil controls (Con) on the left to the highest concentration of oil on the right. Individual genes are the rows. Genes (rows) were hierarchically clustered (Pearson correlation). Higher and lower transcript abundance is indicated in yellow and blue, respectively. Color intensity relates to fold‐increase or decrease of log2 expression (see color scale bar). (B) The first two principal components for the oil exposure‐responsive genes that were conserved in their response between populations. Arrows indicate the trajectory of gene expression change with increasing dose for each population. The base of each arrow represents the no‐oil control condition for that population, wh [file EVA-18-e70112-s002.zip › FigS3.pdf]
