# Supplementary material for: Multiple Stressors in the Anthropocene: Urban Evolutionary History Modifies Sensitivity to the Toxic Effects of Crude Oil Exposure in Killifish
Source: Evol Appl. 2025 May 15;18(5):e70112. doi: 10.1111/eva.70112 (PMC12081835; doi:10.1111/eva.70112)
Supplement: Supplementary file 2 — Appendix S1. List of the 39 polycyclic aromatic compounds, the classification of the PAC (Low molecular weight, LMH or high molecular weight, HMW), the number of ring structures and the carbon numbers. [file EVA-18-e70112-s004.docx]

**Appendix S1.** List of the 39 polycyclic aromatic compounds, the classification of the PAC (Low molecular weight, LMH or high molecular weight, HMW), the number of ring structures and the carbon numbers.

| **Analyte** | **PAC Type Classification** | **Carbon No.** |
| --- | --- | --- |
| Naphthalene | LMW: 2-ring | 10 |
| C2-Naphthalenes | LMW: 2-ring | 12 |
| C3-Naphthalenes | LMW: 2-ring | 13 |
| C4-Naphthalenes | LMW: 2-ring | 14 |
| Acenaphthene | LMW: 3-ring | 12 |
| Acenaphthylene | LMW: 3-ring | 12 |
| Biphenyl | LMW: 2-ring | 12 |
| Dibenzothiophene | LMW: 3-ring | 12 |
| C1-Dibenzothiophenes | LMW: 3-ring | 13 |
| C2-Dibenzothiophenes | LMW: 3-ring | 14 |
| C3-Dibenzothiophenes | LMW: 3-ring | 15 |
| C4-Dibenzothiophenes | LMW: 3-ring | 16 |
| Fluorene | LMW: 3-ring | 13 |
| C1-Fluorenes | LMW: 3-ring | 14 |
| C2-Fluorenes | LMW: 3-ring | 15 |
| C3-Fluorenes | LMW: 3-ring | 16 |
| Anthracene | LMW: 3-ring | 14 |
| Phenanthrene | LMW: 3-ring | 14 |
| C1-Phenanthrenes/Anthracenes | LMW: 3-ring | 15 |
| C2-Phenanthrenes/Anthracenes | LMW: 3-ring | 16 |
| C3-Phenanthrenes/Anthracenes | LMW: 3-ring | 17 |
| C4-Phenanthrenes/Anthracenes | LMW: 3-ring | 18 |
| Fluoranthene | HMW: 4-ring | 16 |
| Pyrene | HMW: 4-ring | 16 |
| C1-Fluoranthenes/Pyrenes | HMW: 4-ring | 17 |
| C2-Fluoranthenes/Pyrenes | HMW: 4-ring | 18 |
| C3-Fluoranthenes/Pyrenes | HMW: 4-ring | 19 |
| C4-Fluoranthenes/Pyrenes | HMW: 4-ring | 20 |
| Chrysene | HMW: 4-ring | 18 |
| C1-Chrysenes | HMW: 4-ring | 19 |
| C2-Chrysenes | HMW: 4-ring | 20 |
| C3-Chrysenes | HMW: 4-ring | 21 |
| C4-Chrysenes | HMW: 4-ring | 22 |
| Benzo(b)fluoranthene | HMW: 5-ring | 20 |
| Benzo(a)pyrene | HMW: 5-ring | 20 |
| Benzo(e)pyrene | HMW: 5-ring | 20 |
| Perylene | HMW: 5-ring | 20 |
| Benzo(g,h,i)perylene | HMW: 6-ring | 22 |
| Indeno(1,2,3-cd)pyrene | HMW: 6-ring | 22 |
